# Histological architecture of the intersphincteric region of the anal canal: implications for the anatomical basis of anal fistula pathways

**DOI:** 10.1007/s00384-026-05123-9

**Published:** 2026-03-27

**Authors:** Satoru Muro, Yasuo Nakajima, Akimoto Nimura, Keiichi Akita

**Affiliations:** 1 Department of Clinical Anatomy, Graduate School of Medical and Dental Sciences, Institute of Science Tokyo, 1-5-45 Yushima, Bunkyo-Ku, Tokyo, 113-8510 Japan; 2 Department of Colorectal Surgery, Moriyama Memorial Hospital, Tokyo, Japan; 3 Department of Functional Joint Anatomy, Biomedical Engineering Laboratory, Institute of Industry Incubation, Institute of Science Tokyo, Tokyo, Japan

**Keywords:** Anal fistula, Parks classification, Intersphincteric space, Longitudinal muscle, Histology

## Abstract

**Purpose:**

To clarify the histological architecture of the intersphincteric region of the anal canal by delineating the layer-specific organization and spatial relationships among the anal sphincter complex and associated muscular and connective tissue components.

**Methods:**

Tissue blocks containing the lateral wall of the anal canal were obtained from 11 adult human cadavers donated for anatomical research. Specimens were examined using descriptive histological and immunohistochemical analyses in transverse and coronal planes. The internal and external anal sphincters, longitudinal muscle, levator ani, interbundle gaps, and connective tissue compartments were identified and analyzed with respect to their three-dimensional organization.

**Results:**

The intersphincteric region exhibited a heterogeneous and layered architecture rather than a uniform plane. The longitudinal muscle demonstrated a mosaic organization consisting of dense and loose components. The dense component terminated near the mid-height of the internal anal sphincter (mean, 54% of its length), whereas the loose component expanded inferiorly and formed a spacious compartment characterized by sparse smooth muscle fibers and loose connective tissue. Inferiorly, loose longitudinal muscle fibers branched and traversed natural interbundle gaps within the external anal sphincter. In addition, two partially overlapping layers of the levator ani were consistently observed, with interposed gaps contributing to the structural complexity of the intersphincteric region.

**Conclusion:**

The intersphincteric region of the anal canal is a structurally complex and compartmentalized anatomical entity. Its heterogeneous histological architecture provides an anatomical substrate that may explain the initiation and directional spread of anal fistulas, including pathways described in classical fistula classifications.

**Supplementary Information:**

The online version contains supplementary material available at 10.1007/s00384-026-05123-9.

## Introduction

Anal fistulas are frequently encountered in daily colorectal practice and remain therapeutically challenging because of their high recurrence rates and the risk of postoperative fecal incontinence [1, 2]. Successful management of anal fistulas relies on a precise understanding of the underlying anatomy of the anal canal and sphincter complex. Although several classification systems have been proposed to describe fistula pathways, their clinical utility ultimately depends on how accurately they reflect the actual anatomical structures involved.

Among these systems, the Parks classification remains the most widely used framework for preoperative assessment and surgical decision-making [3, 4]. This classification categorizes fistula tracts into intersphincteric, trans-sphincteric, suprasphincteric, and extrasphincteric types according to their relationship with the internal anal sphincter (IAS) and external anal sphincter (EAS). Despite its long-standing clinical relevance, however, the Parks classification presupposes the existence of an “intersphincteric” region without providing a clear anatomical definition of this term. In the present study, the intersphincteric region is defined anatomically as the area located between the IAS and the EAS, irrespective of its internal histological heterogeneity. In the original descriptions, abscess formation was illustrated as occurring “between the sphincters,” with subsequent upward, downward, or lateral spread, yet it remained unclear whether this space includes the longitudinal muscle (LM), at what level along the anal canal it is located, or which tissue layers—such as muscle bundle clefts or connective tissue compartments—are involved [4].

Even with advances in imaging modalities such as magnetic resonance imaging (MRI) and three-dimensional endoanal ultrasonography (3D-EAUS), the detailed microanatomy of the intersphincteric region remains insufficiently understood [5, 6]. These techniques provide valuable macroscopic information but lack the resolution necessary to delineate the fine histological organization of the LM and adjacent connective tissue structures. Consequently, the intersphincteric region is often interpreted as a simple anatomical plane, despite growing evidence that it represents a more complex anatomical entity.

Previous anatomical and histological studies have highlighted the multilayered architecture of the anal sphincter complex and the presence of spaces between its muscular components [7–9]. Building on these studies, our previous investigations focused on the LM coursing between the IAS and EAS and its relationship with surrounding connective and adipose tissues [10–13]. We demonstrated that the LM is not a uniform, continuous layer but instead exhibits a mosaic organization composed of dense longitudinal smooth muscle bundles and loosely arranged smooth muscle fibers intermingled with loose connective tissue [14]. These observations suggested that the region traditionally labeled as “intersphincteric” encompasses heterogeneous tissue components rather than a single, well-defined plane.

The present study was designed to clarify the histological architecture of the intersphincteric region of the anal canal by systematically analyzing the layer-specific organization and spatial relationships among the IAS, LM, EAS, levator ani (LA), and associated connective tissue compartments. By defining the structural heterogeneity of this region on a histological basis, we further sought to explore how these anatomical features may provide a substrate for the initiation and propagation of anal fistulas, including pathways described in the Parks classification.

## Methods

### Study design

This was a descriptive anatomical study of the anal canal based on histological and immunohistochemical analyses of formalin-fixed cadaveric specimens. The primary objective was to delineate the histological architecture and layer-specific organization of the intersphincteric region by examining the spatial relationships among the IAS, LM, EAS, LA, and intervening connective tissue compartments.

### Cadaver specimens

Eleven adult cadavers (five males and six females; age at death, 58–91 years; mean, 80.6 years) were included in this study. All specimens were obtained through the body donation program of the Department of Anatomy at our institution in accordance with the Japanese Act on Body Donation for Medical and Dental Education. Donation was based on the donors’ documented wishes prior to death.

Cadavers with a known history of anorectal disease, previous anorectal surgery, or any apparent degeneration or injury affecting the anal sphincter complex or adjacent structures were excluded. All bodies were fixed by arterial perfusion with 8% formalin and subsequently stored in 30% alcohol to prevent fungal growth while maintaining tissue pliability.

### Tissue sampling

The pelvis was obtained en bloc from each cadaver and divided along the midsagittal plane using a diamond saw. From each hemipelvis, the lateral wall of the anal canal and surrounding tissues was excised as a single tissue block, including the IAS, LM, EAS, subcutaneous tissues, and portions of the ischioanal fossa (IAF).

For histological evaluation, tissue blocks were trimmed to include the full longitudinal extent of the anal canal, from above the anorectal junction to the anal verge. Sections were prepared in the transverse plane (perpendicular to the long axis of the anal canal) and the coronal plane (parallel to the long axis), allowing multidirectional assessment of intermuscular spaces and the course of the LM.

### Histological processing

All tissue blocks were embedded in paraffin and serially sectioned into 5-μm-thick slices at 1-mm intervals. Coronal blocks were processed in the same manner. Sections were stained with hematoxylin and eosin, Masson’s trichrome, and Elastica van Gieson to visualize the overall architecture of muscle bundles and connective tissue components.

Immunohistochemical analyses were performed when required using antibodies against smooth muscle actin (Ready-to-Use Actin, Smooth Muscle Ab-1, Clone 1A4; Thermo Fisher Scientific, Fremont, CA, USA) and skeletal muscle myosin (Ready-to-Use Myosin, Skeletal Muscle Ab-2, Clone MYSN02; Thermo Fisher Scientific) to distinguish smooth muscle fibers from skeletal muscle fibers within the LM layer and sphincter complex.

### Microscopic analysis

Microscopic examination was performed at magnifications ranging from ×40 to ×400 to evaluate the detailed microarchitecture of the lateral wall of the anal canal. The mucosa, submucosa, and Treitz muscle were first identified, followed by assessment of the thickness, arrangement, and spatial relationships of the IAS and EAS muscle bundles.

Particular attention was directed to the LM layer located between the IAS and EAS. Based on our previous findings, this layer was classified into dense and loose components composed of compact smooth muscle bundles and sparsely arranged smooth muscle fibers intermingled with loose connective tissue, respectively [14]. The Treitz muscle was identified according to established anatomical criteria [15], and the components of the LA muscle were evaluated based on prior detailed anatomical descriptions [16].

The distribution of intermuscular spaces, loose connective tissue, and adipose tissue around the LM and between the IAS and EAS was examined, with special emphasis on changes in these structures along the longitudinal axis of the anal canal. The branching patterns and outward course of LM fibers traversing interbundle gaps within the EAS toward the subcutaneous tissue and IAF were also documented.

Observations obtained from transverse and coronal sections were integrated to construct a schematic representation of the sphincteric layers and the histological organization of the intersphincteric region.

### Morphometric analysis

For quantitative assessment, coronal sections in which the entire cranio-caudal length of the IAS was included within a single histological section were selected (*n* = 8). Whole-slide images were acquired using a virtual slide scanner (NanoZoomer-SQ, Hamamatsu Photonics, Japan), and measurements were performed using the associated viewing software (NDP.view2, Hamamatsu Photonics).

The IAS length was defined as the distance from the superior to the inferior margin along the cranio-caudal axis in coronal sections. The endpoint of the dense LM was measured as the distance from the superior IAS margin to the most distal level at which dense LM fibers were continuously identified. The relative endpoint was expressed as a percentage of the total IAS length.

For positional analysis, the IAS length of each specimen was normalized from 0% (superior margin) to 100% (inferior margin), and measurements were obtained at 10% intervals. At each level, the thickness of dense and loose LM components was measured perpendicular to the IAS axis. Relative proportions were calculated as Dense% = Dense/(Dense + Loose) and Loose% = Loose/(Dense + Loose).

### Ethical considerations

This study was approved by the Institutional Review Board of the Institute of Science Tokyo (approval no. M2018-006). All cadavers were handled anonymously and in strict accordance with the donors’ wishes.

## Results

**Fig. 1:**
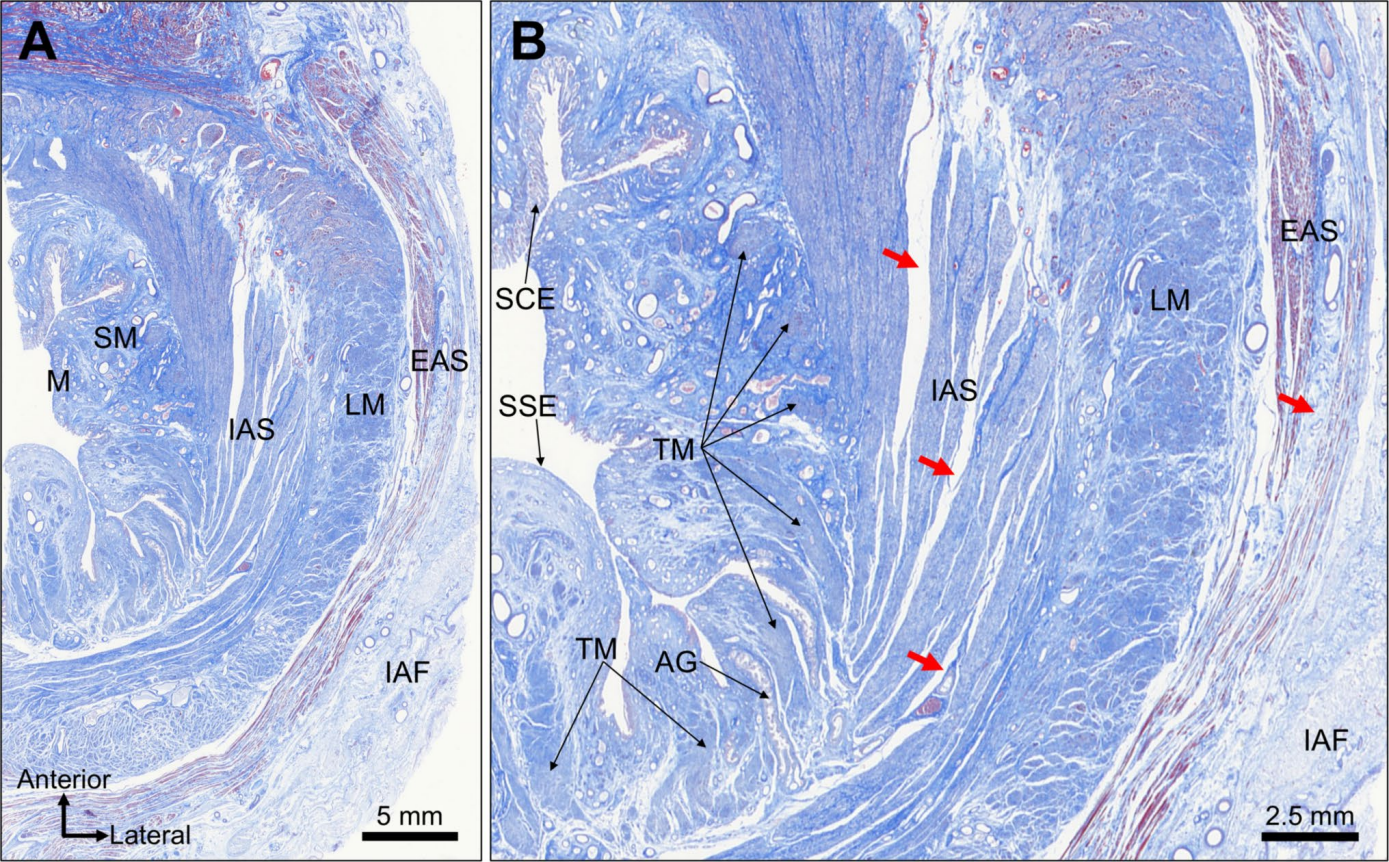
Transverse histological sections at the dentate line level. **A** Low-magnification transverse section demonstrating the mucosa (M), submucosa (SM), and muscular layers composed of the internal anal sphincter (IAS), longitudinal muscle (LM), and external anal sphincter (EAS). The fat of the ischioanal fossa (IAF) was visible lateral to the EAS. **B** High-magnification view of the transitional epithelium showing a mixture of simple columnar epithelium (SCE) and stratified squamous epithelium (SSE). The SM contains anal glands (AGs) and the Treitz muscle (TM). Red arrows highlight the gaps in the muscle bundles within the IAS and EAS. The LM occupies the intersphincteric region, with its fibers oriented longitudinally along the anal canal axis

The mucosal, submucosal, and muscular layers of the anal canal were clearly identified in transverse sections at the level of the dentate line. Within the muscular layer, the IAS, LM, and EAS were observed from the inner to outer aspects (Fig. 1A). Adipose tissue was present in the IAF lateral to the EAS. Under high magnification, the mucosa showed a mixture of simple columnar and stratified squamous epithelium, thus confirming that the specimens represented the transitional zone adjacent to the dentate line (Fig. 1B). The submucosa contains the anal glands and Treitz muscle. The IAS and EAS consisted of circularly oriented muscle bundles with discernible interbundle gaps (Fig. 1B). The LM occupied the intersphincteric region between the IAS and EAS and showed a longitudinal fiber orientation aligned with the anal canal axis.

**Fig. 2:**
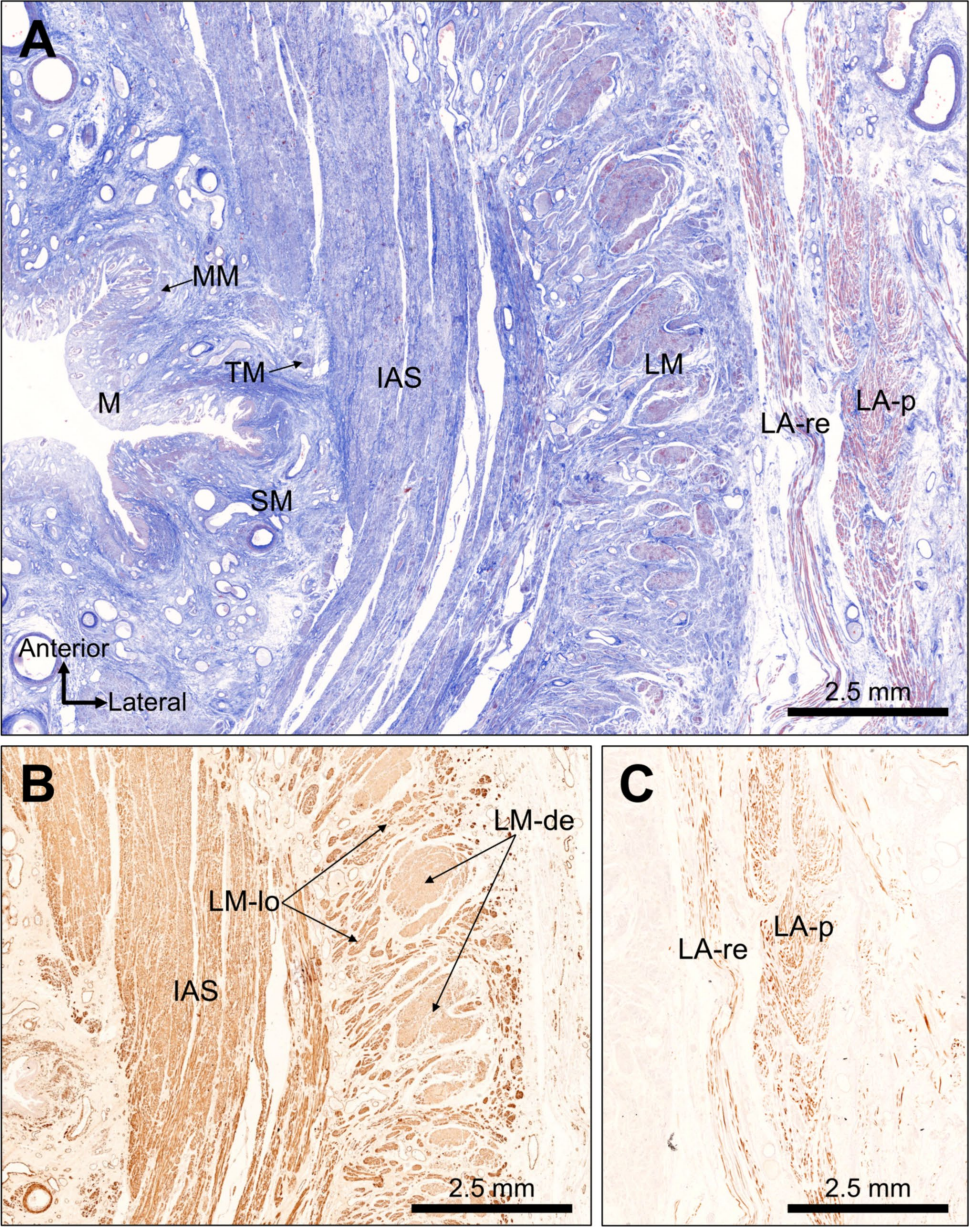
Transverse histological sections of the upper anal canal. **A** Simple columnar epithelium with underlying lamina propria and muscularis mucosae (MM). The Treitz muscle (TM) lies immediately internal to the internal anal sphincter (IAS). The muscular layer consists of the IAS, the longitudinal muscle (LM), and the levator ani (LA). Based on established anatomical criteria, the LA was identified as an inner bundle attached to the rectum (LA-re) and an outer bundle encircling the posterior anal canal (LA-p). **B** Smooth muscle immunostaining showing dense LM (LM-de) and loose LM (LM-lo). LM-de appeared as aligned round bundles surrounded by LM-lo fibers. **C** Skeletal muscle immunostaining confirmed the two-layered configuration of the LA (LA-re and LA-p)

In transverse sections from the upper anal canal, the lamina propria and muscularis mucosae were identified beneath the simple columnar epithelium, and the Treitz muscle was located immediately inside the IAS (Fig. 2A). From the luminal side outward, the muscular layer consists of the IAS, LM, and LA (Fig. 2B, C). The LA appeared as two distinct layers of skeletal muscle. Based on established anatomical criteria, these were designated the inner rectal-attached bundle (LA-re) and the outer posterior encircling bundle (LA-p). The two-layered structure of LA was confirmed by immunostaining for skeletal muscle myosin (Fig. 2C). Immunohistochemistry for smooth muscle actin showed that the LM comprised densely packed smooth muscle bundles (LM-de) and loosely arranged fibers interspersed with connective tissue (LM-lo) (Fig. 2B). In the cross-section, dense LM bundles appeared as aligned, round profiles surrounded by loose LM fibers.

**Fig. 3:**
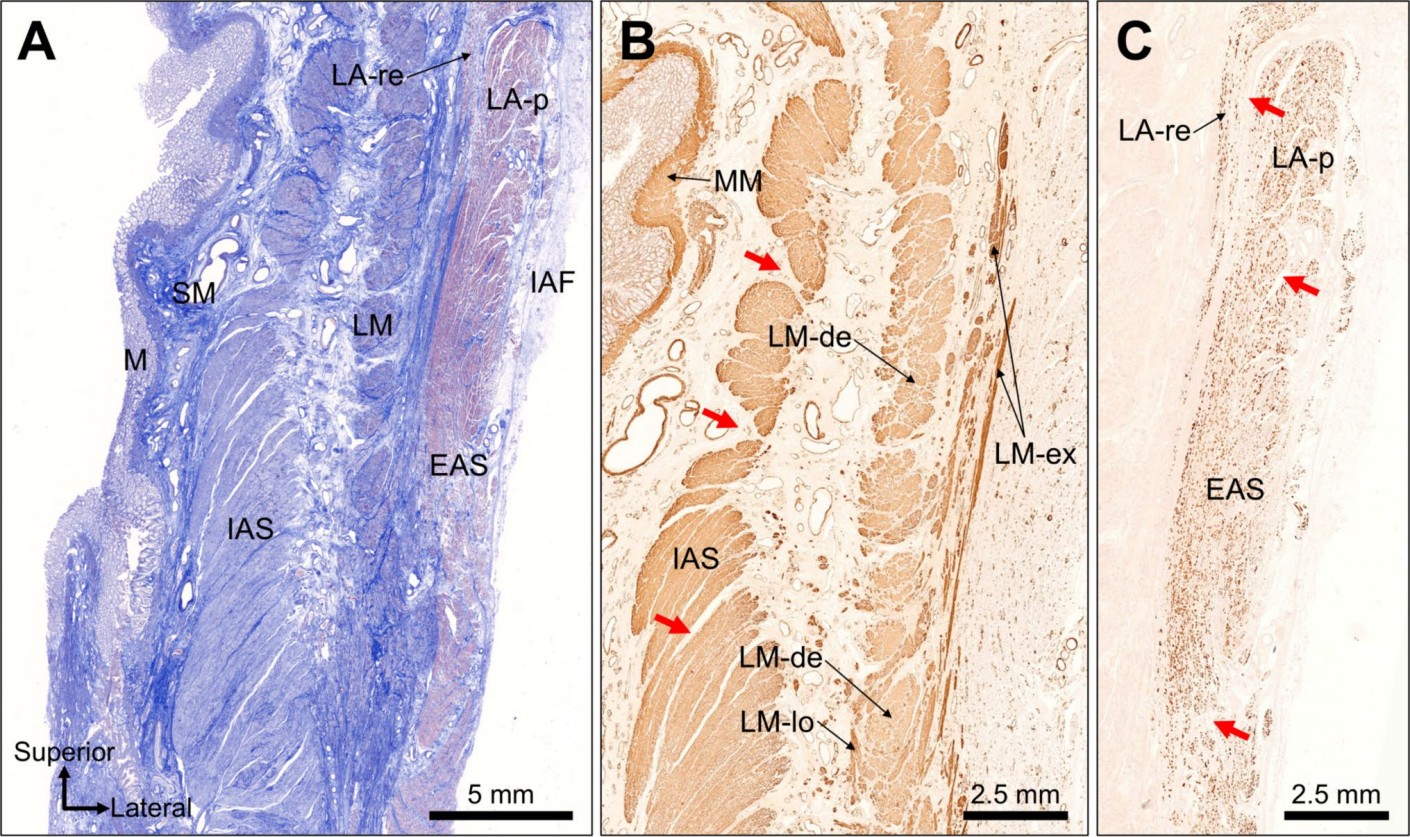
Coronal histological sections of the upper anal canal wall. **A** Low-magnification coronal sections identifying mucosa (M), submucosa (SM), internal anal sphincter (IAS), longitudinal muscle (LM), external anal sphincter (EAS), levator ani (LA), and ischioanal fossa (IAF) fat. **B** Smooth muscle immunostaining showing focal interbundle gaps within the IAS (red arrows). Dense and loose LM components are present, with portions of loose LM projecting superolaterally as extension fibers (LM-ex). **C** Skeletal muscle immunostaining demonstrating the layered arrangement of the LA: the LA-re descends medially to the LA-p and enters the intersphincteric region. Red arrows indicate gaps between the LA-re and LA-p and within EAS bundles. A comparison of the stains shows LM-ex fibers enveloping and directly attaching to LA-re as they enter the intersphincteric space

Coronal sections from the upper anal canal wall showed the mucosa, submucosa, IAS, LM, EAS, LA, and ischioanal fat (Fig. 3A). Smooth muscle immunostaining revealed focal gaps between the IAS muscle bundles (Fig. 3B). Dense and loose LM regions were evident, and portions of the loose LM extended laterally and superiorly, projecting into the smooth muscle fibers (LM-ex). In contrast, skeletal muscle immunostaining delineated the layered configuration of the LA: the LA-re descended from the superior aspect, passed medially to the LA-p, and entered the intersphincteric region. Distinct gaps were present between the LA-re and LA-p, as well as between the EAS muscle bundles (Fig. 3C). A comparison of smooth and skeletal muscle staining showed that the projecting smooth muscle fibers (LM-ex) were enveloped by and directly attached to the LA-re fibers entering the intersphincteric region from above.

**Fig. 4:**
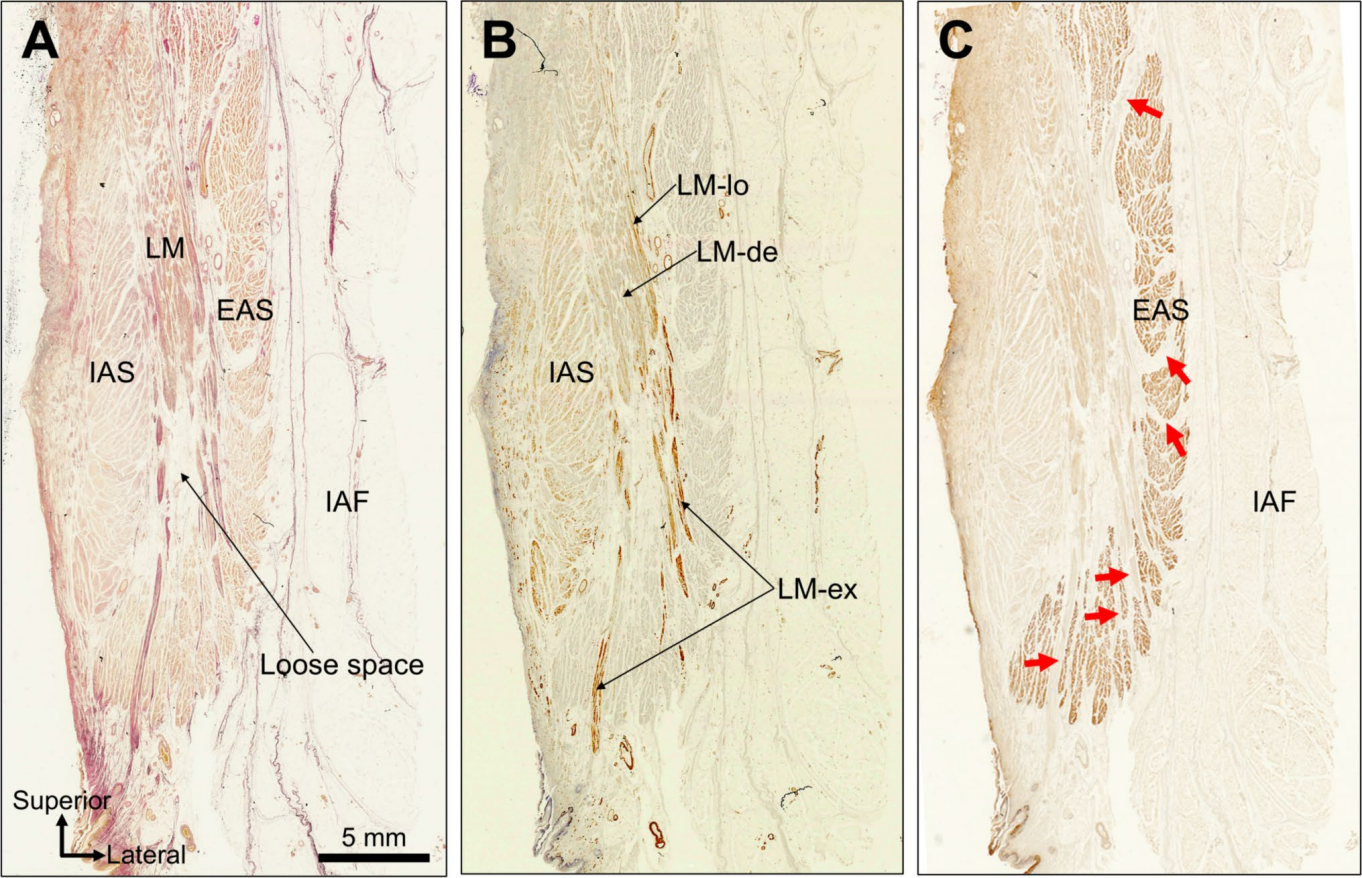
Coronal histological sections of the lower anal canal. **A** Identification of the internal anal sphincter (IAS), longitudinal muscle (LM), external anal sphincter (EAS), and ischioanal fossa (IAF) fat. A prominent loose compartment composed of loose LM (LM-lo) fibers and loose connective tissue occupies the inferior portion of the LM. **B** Smooth muscle immunostaining demonstrates termination of the dense LM at approximately mid-IAS height. Below this level, the loose LM expands into a branching network that traverses the gaps between the EAS bundles and extends toward the subcutaneous fat. **C** Skeletal muscle immunostaining shows the EAS muscle bundles. Red arrows indicate gaps within the EAS bundles

The IAS, LM, EAS, and ischioanal fat were identified in coronal sections of the lower anal canal (Fig. 4A). Smooth muscle immunostaining revealed that the dense LM terminated approximately midway from the height of the IAS, below which the loose LM expanded and formed branching extensions. The loose LM fibers coursed between the EAS bundles and extended toward the subcutaneous fat (Fig. 4B, C). The sites where the loose LM penetrated the EAS were aligned with the natural interbundle gaps of the EAS (Fig. 4C). In the inferior portion of the LM, a spacious compartment consisting of loose LM fibers and connective tissue was consistently present. This inferior loose space was identified in all 11 specimens (11/11, 100%).

**Fig. 5:**
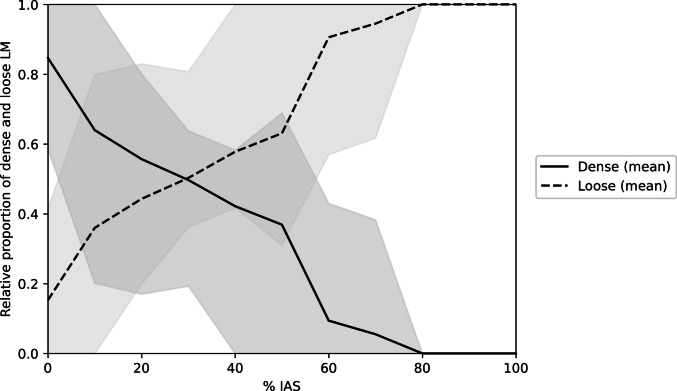
Vertical transition of dense and loose longitudinal muscle components along the internal anal sphincter. The IAS length was normalized to 0–100% in each specimen (*n* = 8). The relative proportions of the dense and loose LM components were calculated at 10% intervals along the normalized IAS length. The solid line represents the mean relative proportion of the dense LM, and the dashed line represents the mean relative proportion of the loose LM (calculated as 1 − dense proportion). Shaded areas indicate the minimum–maximum range across specimens at each level. The dense LM predominated in the superior portion of the IAS and progressively decreased toward the inferior side, whereas the loose LM showed the opposite pattern, becoming the dominant component in the inferior region

To quantitatively assess this vertical transition, morphometric measurements were performed in eight coronal specimens (Table 1). The endpoint of the dense LM was located at a mean of 54.0% of the IAS length (range, 33.6–75.7%), indicating that the dense component was primarily confined to the superior half of the IAS height. Positional analysis at 10% intervals of the normalized IAS length demonstrated a reciprocal shift in the relative proportions of dense and loose LM toward the inferior aspect (Fig. 5; Supplementary Table S1). At the superior margin (0% IAS), the dense LM accounted for a mean of 84.7%, whereas the loose LM comprised 15.3%. With increasing distance toward the inferior margin, the dense component progressively decreased (36.9% at 50% IAS) and became negligible at the 80–100% IAS levels. Conversely, the loose LM proportion increased correspondingly, becoming the predominant component in the inferior half of the IAS and approaching 100% at the 80–100% IAS levels.

**Table 1:** Morphometric parameters of the intersphincteric region (*n* = 8)

Parameter	Mean (range)
IAS maximum thickness (mm)	**3.47 (2.42–5.01)**
EAS maximum thickness (mm)	**4.17 (2.77–5.78)**
Intersphincteric region maximum thickness (mm)	**3.67 (2.54–4.82)**
IAS length (mm)	**27.91 (23.4–31.9)**
Dense LM relative endpoint (% of IAS length)	**54.0 (34.6–75.7)**

## Discussion

**Fig. 6:**
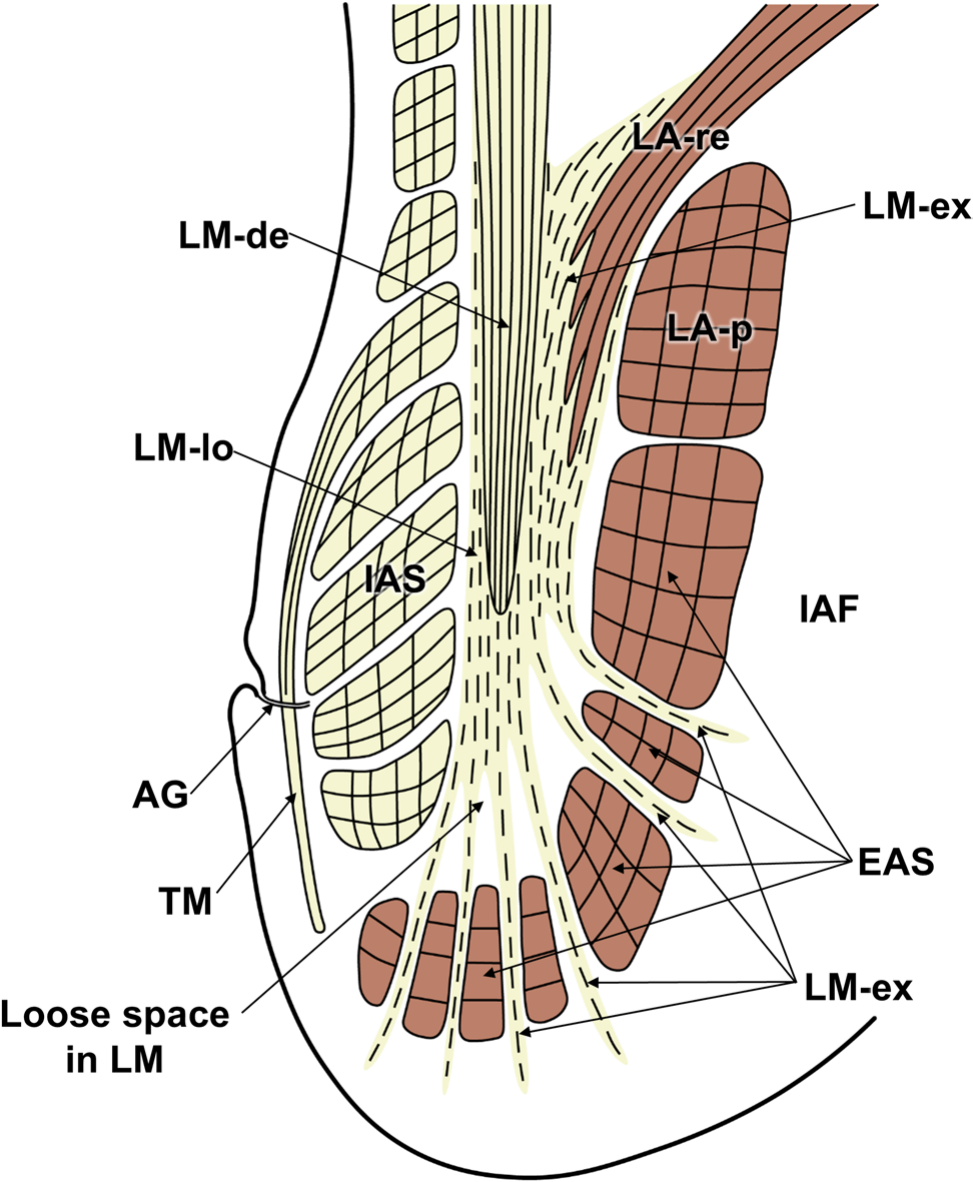
Integrated schematic reconstruction of the intersphincteric region. The key anatomical features illustrated include the following: (1) interbundle gaps within the internal anal sphincter (IAS), external anal sphincter (EAS), and levator ani (LA); (2) loose space in the inferior longitudinal muscle (LM); (3) overlapping configuration of the LA inner bundle attached to the rectum (LA-re) and the LA outer bundle encircling the posterior anal canal (LA-p), with LA-re extending into the intersphincteric space

This study provides a detailed histological characterization of the intersphincteric region of the anal canal based on an integrated analysis of transverse and coronal sections. As summarized schematically in Fig. 6, the intersphincteric region was shown to be a structurally heterogeneous and compartmentalized anatomical entity rather than a simple plane. Key structural features included the presence of interbundle gaps within the IAS, EAS, and LA, as well as a complex spatial relationship among multiple muscular layers and intervening connective tissue components. In addition, the LA was composed of two partially overlapping layers, with the inner component (LA-re) extending into the intersphincteric region and separated from the outer component (LA-p) by a distinct gap.

A major anatomical finding of this study was the layer-specific organization of the LM. The dense LM component terminated at approximately the mid-height of the IAS, whereas the loose LM component expanded inferiorly, branched along the longitudinal axis of the anal canal, and traversed natural interbundle gaps within the EAS toward the subcutaneous tissue and IAF. Quantitative analysis supported this vertical stratification, demonstrating that the dense LM endpoint was located at a mean of 54% of the IAS length and that the relative proportion of dense LM progressively decreased toward the inferior margin, with reciprocal predominance of the loose component. Furthermore, lateral–superior extensions of loose LM fibers were observed to attach around the descending LA-re fibers. Importantly, this configuration indicated that the interface between the LM and EAS does not exhibit direct anatomical continuity with the supralevator compartment.

**Fig. 7:**
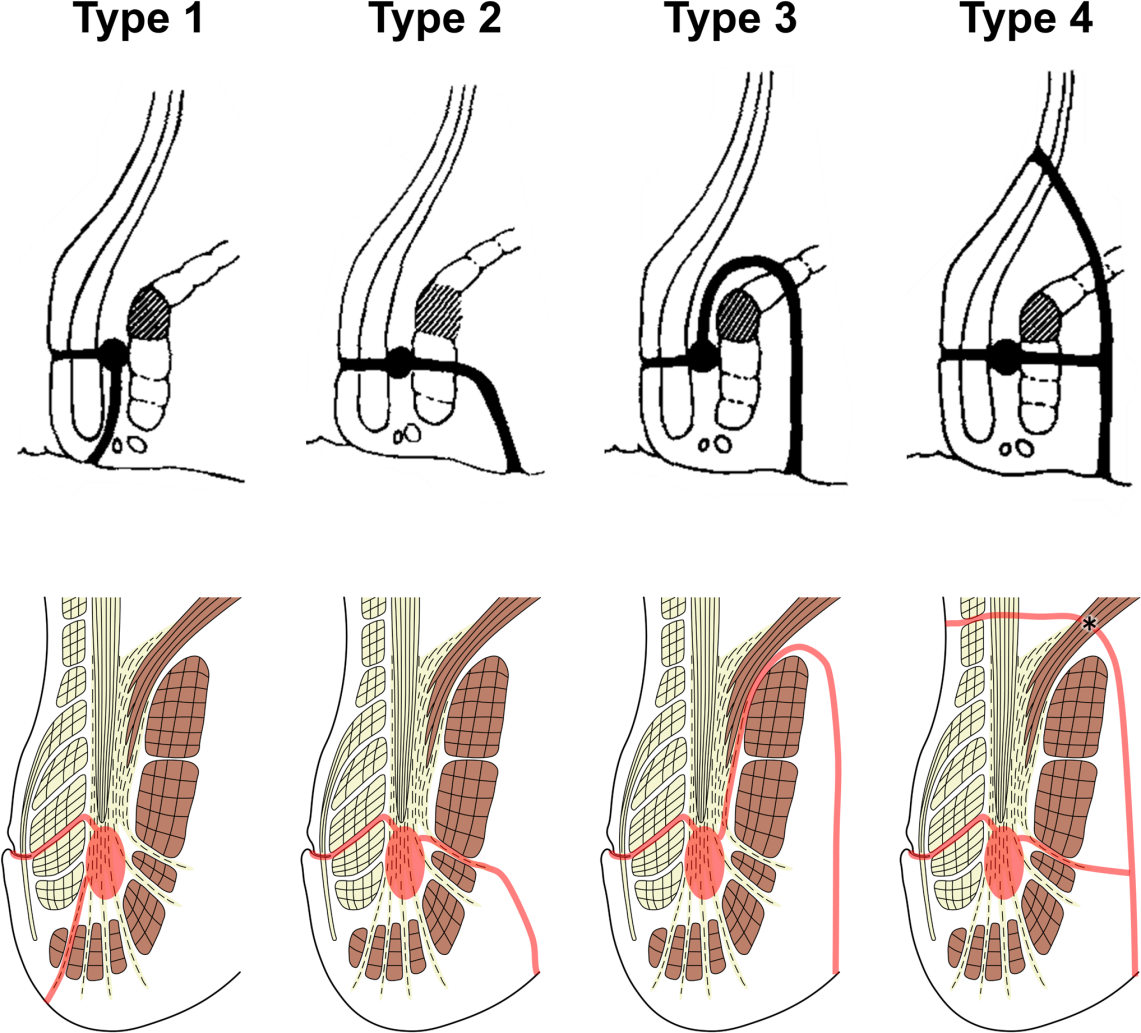
Schematic interpretation of the histological architecture of the intersphincteric region in relation to the Parks classification of anal fistulas. This diagram integrates the histologically identified structures of the internal anal sphincter (IAS), longitudinal muscle (LM), external anal sphincter (EAS), and levator ani (LA), with particular emphasis on the loose smooth muscle compartment in the inferior portion of the LM. The region traditionally illustrated by Parks as the site of an “intersphincteric abscess” is shown to correspond anatomically to this inferior loose LM compartment. Natural interbundle gaps within the IAS and EAS, as well as the gap between the inner (LA-re) and outer (LA-p) components of the levator ani, are depicted as structural continuities that may account for the directional extension patterns described in the Parks classification

When these histological findings are interpreted in the context of the Parks classification, the region traditionally illustrated as the site of an “intersphincteric abscess” corresponds closely to the loose smooth muscle space located in the inferior portion of the LM (Fig. 7). In the surgical literature, this area has been described as the “intersphincteric space”; however, based on our histological observations, we consider it to represent a structurally complex intersphincteric region rather than a simple space. It should be emphasized that this correspondence represents a reasoned anatomical inference based on observations in normal specimens, rather than direct evidence derived from pathological fistula tissues. The four fistula types described by Parks have been used clinically for more than five decades; however, the original descriptions did not provide a detailed definition of the histological nature of the “intersphincteric space” [4]. Earlier anatomical and histological studies primarily focused on the sphincter muscles themselves and did not sufficiently address the internal architecture of the LM situated between the IAS and EAS, thereby underestimating the structural complexity of this region [7–9, 17]. In addition, neural elements within the intersphincteric region have been shown to connect with the enteric plexus of the rectum and to contain mixed autonomic components [18], further supporting the concept that this area represents a structurally complex anatomical entity rather than a simple anatomical plane.

From a histological perspective, the loose inferior compartment of the LM represents a low-resistance space composed of sparse smooth muscle fibers and loose connective tissue. This structural characteristic is consistent with recent evidence indicating that cryptoglandular infections preferentially spread along pre-existing low-resistance connective tissue pathways [19]. Based on the present observations, fistula pathways described in the Parks classification can be interpreted as extensions along anatomically definable structures, such as the interbundle gaps within the EAS, the gap between LA-re and LA-p, and the branching loose components of the LM. In contrast, although type IV fistulas are described as extending into the supralevator space, a distinct pre-existing anatomical continuity corresponding to such an extension was not clearly identified in the normal specimens examined in this study. This finding suggests that supralevator spread may depend on pathological disruption, secondary inflammatory changes, or structural alterations beyond normal anatomical architecture.

The present histological redefinition of the intersphincteric region has important clinical implications. This region has traditionally been regarded as a homogeneous plane, an oversimplification that has been implicated in interpretive inconsistencies in EAUS and MRI, as well as in discrepancies among fistula classification systems [20–22]. A shared limitation of multiple classification schemes, including more recent imaging-guided systems, is the absence of a detailed histological foundation for the intersphincteric space [23]. Recognition of the dense and loose components of the LM and their 3D arrangement may help explain the heterogeneous imaging signals observed in this region, consistent with MRI-based findings suggesting that dense and loose LM components can be distinguished [14]. Moreover, sex-related differences in the course of intersphincteric fistulas reported in 3D-EAUS studies further support the notion that this region is anatomically complex and heterogeneous rather than uniform [24]. The present quantitative measurements of IAS, EAS, and intersphincteric thickness provide reference values obtained without endoluminal distortion, which may facilitate comparison with MRI-based measurements and help interpret discrepancies observed with EAUS. Although the concept of the intersphincteric pathway is well established in colorectal surgery and radiology, this study does not seek to redefine this clinical paradigm but rather to provide a high-resolution histological framework that refines and substantiates these established observations.

Some limitations of this study should be acknowledged. First, all specimens were obtained from formalin-fixed cadavers of older adult donors, and tissue characteristics such as firmness and fat content may differ from those of living individuals. Age-related changes in anal canal tissues, including muscle atrophy, increased connective tissue deposition, and alterations in fat distribution, may influence the absolute thickness and apparent density of muscular components. In particular, age-associated muscle atrophy could potentially accentuate the relative prominence of loose connective tissue compartments, thereby affecting the perceived extent of the inferior loose space. Because cryptoglandular fistulas most commonly affect middle-aged adults, it remains possible that the relative proportions of dense and loose LM components, as well as the width of interbundle gaps, may differ in younger individuals. Further histological studies, including younger specimens, are necessary to determine the extent to which these findings are preserved across age groups. Second, the analysis was limited to the lateral wall of the anal canal, and regional variations in the anterior or posterior walls were not assessed. The anterior wall, particularly in females, contains additional structures such as the perineal body and vaginal wall, which may influence the organization of the intersphincteric region. Likewise, posteriorly, the relationship with the anococcygeal ligament and surrounding connective tissues may introduce structural variations. Therefore, the conclusions of this study should be interpreted as primarily applicable to the lateral wall, and direct extrapolation to the entire circumferential anatomy of the anal canal should be made with caution. Future investigations examining anterior and posterior regions are warranted to determine whether the layer-specific organization observed laterally is consistently preserved throughout the anal canal. Third, the correspondence between histological structures and the Parks classification was inferential and was not directly validated using specimens from patients with active fistulas. Fourth, although neural structures are known to play important roles in continence and sensory function, this study focused primarily on the muscular and connective tissue architecture of the intersphincteric region, and specific immunohistochemical evaluation of neural elements was not performed. Finally, because this study was based on normal anatomical specimens, pathways that may arise only through pathological disruption, including supralevator extension, could not be fully evaluated. However, the analysis focused primarily on the spatial organization and layer-specific relationships among anatomical structures rather than on absolute volumetric parameters. The consistent identification of dense and loose LM components and their vertical transition across specimens suggests that the fundamental architectural arrangement is preserved, although quantitative proportions may differ in younger populations. Nevertheless, the structural reinterpretation presented here is consistent with recent imaging and immunological findings, supporting its anatomical and clinical plausibility.

In conclusion, this study demonstrates that the intersphincteric region of the anal canal is a structurally complex and heterogeneous anatomical entity composed of multiple muscle layers, interbundle gaps, and loose connective tissue compartments. The identification of a loose inferior compartment within the LM provides a high-resolution histological framework that helps refine the pathways described in the Parks classification, particularly those related to the “intersphincteric abscess,” which can be interpreted as routes of least resistance. A clearer understanding of the 3D histological architecture of this region may improve the interpretation of EAUS and MRI findings, enhance consistency among classification systems, and ultimately contribute to more informed preoperative evaluation and sphincter-preserving surgical strategies.

## Supplementary Information

Below is the link to the electronic supplementary material.

ESM1 Supplementary Table S1. Relative proportion of dense and loose longitudinal muscle (LM) along the normalized IAS length (DOCX 22.5 KB)

## Data Availability

The data that support the findings of this study are available from the corresponding author upon reasonable request.
