# Supplementary material for: Histological architecture of the intersphincteric region of the anal canal: implications for the anatomical basis of anal fistula pathways
Source: Int J Colorectal Dis. 2026 Mar 27;41(1):83. doi: 10.1007/s00384-026-05123-9 (PMC13031206; doi:10.1007/s00384-026-05123-9)
Supplement: Supplementary file 1 — Supplementary Table S1. Relative proportion of dense and loose longitudinal muscle (LM) along the normalized IAS length (DOCX 22.5 KB) [file 384_2026_5123_MOESM1_ESM.docx]

**Supplementary Table S1. Relative proportion of dense and loose longitudinal muscle (LM) along the normalized IAS length**

| %IAS | Dense mean (%) | Dense range (%) | Loose mean (%) | Loose range (%) |
| --- | --- | --- | --- | --- |
| 0 | 84.7 | 58.5–100.0 | 15.3 | 0.0–41.5 |
| 10 | 64.0 | 20.1–100.0 | 36.0 | 0.0–79.9 |
| 20 | 55.7 | 17.0–80.1 | 44.3 | 19.9–83.0 |
| 30 | 49.7 | 19.3–63.8 | 50.3 | 36.2–80.7 |
| 40 | 42.2 | 0.0–58.3 | 57.8 | 41.7–100.0 |
| 50 | 36.9 | 0.0–68.9 | 63.1 | 31.1–100.0 |
| 60 | 9.4 | 0.0–43.0 | 90.6 | 57.0–100.0 |
| 70 | 5.5 | 0.0–38.3 | 94.5 | 61.7–100.0 |
| 80 | 0.0 | 0.0–0.0 | 100.0 | 100.0–100.0 |
| 90 | 0.0 | 0.0–0.0 | 100.0 | 100.0–100.0 |
| 100 | 0.0 | 0.0–0.0 | 100.0 | 100.0–100.0 |

The internal anal sphincter (IAS) length was normalized from 0% (superior margin) to 100% (inferior margin) in each specimen (n = 8), and measurements were obtained at 10% intervals. Values represent the mean and range (minimum–maximum) of the relative proportions of dense and loose LM at each level. Relative proportions were calculated from direct measurements of dense and loose LM thickness at each level:

Dense% = Dense / [Dense + Loose]; Loose% = Loose / [Dense + Loose]
